# Social Media Use and Openness to “Sugar Relationships”: Cultural Tightness–Looseness as a Moderator Across 22 Countries

**DOI:** 10.1007/s10508-026-03551-w

**Published:** 2026-10-22

**Authors:** Norbert Meskó, Jessica S. Ehlers, Anna Molnár, Hyemin Han

**Affiliations:** 1 Department of Cognitive and Evolutionary Psychology, Faculty of Humanities and Social Sciences, Institute of Psychology, University of Pécs, Ifjúság Utca 6., Pécs, 7624 Hungary; 2 Educational Psychology Program, University of Alabama, Tuscaloosa, AL USA

**Keywords:** Social media, Sugar Relationships, Sexual-economic exchange, Cultural tightness–looseness, Cross-country comparison

## Abstract

Social media use has been widely linked to sexual attitudes, yet it remains unclear whether its association with openness to sugar relationships is uniform across countries or contingent on country-level normative contexts. In the present study, we examined whether country-level cultural tightness–looseness moderated the relationship between social media use and openness to sugar relationships across 22 countries. Openness to sugar relationships was conceptualized as a manifestation of a broader orientation toward sexual–economic exchange. Using data from 22,292 adult women drawn from a large cross-national dataset, we tested this framework with Bayesian multilevel modeling. Measurement invariance of the openness construct was assessed, and alignment procedures were applied to address cross-country non-invariance prior to analysis. Cultural tightness–looseness was modeled as a country-level moderator of the individual-level association between social media use and openness to sugar relationships, while age was included as a control variable. Results revealed a positive association between social media use and openness to sugar relationships. In addition, this association was modestly stronger in countries characterized by lower normative constraint, suggesting that country-level norm strength may shape the attitudinal relevance of digital exposure. Models including this interaction outperformed alternative specifications that incorporated a range of theoretically plausible country-level covariates, including indicators of digital infrastructure, socioeconomic structure, and internet freedom. Although the interaction effect size was modest, the findings suggest that the attitudinal correlates of social media use vary systematically across countries and highlight the importance of country-level normative contexts in research on sexual–economic openness.

**Supplementary Information:**

The online version contains supplementary material available at https://doi.org/10.1007/s10508-026-03551-w.

## Introduction

Digital platforms have become central environments for the formation and circulation of sexual norms, intimate scripts, and relationship expectations (Wang, 2023). Beyond providing access to information, social media and other online spaces are associated with how sexual behaviors are interpreted, evaluated, and normalized, particularly among younger individuals, who are more extensively immersed in digital environments in which such relationship scripts are more visible and socially negotiated. These effects are typically explained by several interrelated mechanisms. Social media increases exposure to sexualized and relationship-relevant content, amplifies social comparison processes, and contributes to the perception of peer norms, which in turn are associated with sexual attitudes and behavioral intentions (Ahmmad et al., 2025). In particular, online environments may function as a “super-peer” context in which perceived norms are exaggerated and risky or nontraditional behaviors become normalized through repeated exposure and peer endorsement (Cookingham & Ryan, 2015). Systematic reviews and meta-analytic findings further indicate that exposure to sexualized media content is consistently associated with sexual attitudes, perceived peer norms, sexual initiation patterns, and risk-related behaviors, highlighting the role of digital environments in contemporary sexual socialization processes (Brown et al., 2006; Coyne et al., 2019; Fevriasanty et al., 2021; Ward, 2003). These processes are especially pronounced in normatively sensitive domains, where individuals rely more heavily on social cues to assess what forms of intimacy are socially acceptable, desirable, or legitimate.

Importantly, the psychological impact of digital exposure is unlikely to be uniform across countries. Countries differ markedly in the strength of social norms and in the tolerance of behavioral deviance—differences commonly captured by country-level indicators such as cultural tightness–looseness—shaping not only which behaviors are sanctioned or rewarded but also how individuals respond to social information more broadly (Henrich et al., 2010; Markus & Kitayama, 1991). From this perspective, digital exposure does not exert a direct or deterministic influence on intimate attitudes. Instead, its effects are filtered through country-level normative frameworks that regulate whether nontraditional or normatively ambivalent relationship forms are perceived as legitimate and psychologically accessible (Chiu & Cheng, 2010).

One influential framework for capturing such cross-country variation is cultural tightness–looseness, which refers to the degree to which social norms are clearly defined, strongly enforced, and deviations are sanctioned within a country (Uz, 2014). In countries characterized by higher cultural tightness, norm violations tend to carry higher social costs, whereas countries characterized by lower cultural tightness allow greater behavioral latitude and display higher tolerance for unconventional practices (Gelfand et al., 2011). Crucially, cultural tightness–looseness does not prescribe specific preferences or behaviors. Rather, it structures the conditions under which social information becomes psychologically actionable. In countries characterized by lower normative constraint, social comparison processes and socially transmitted cues are more readily engaged, increasing the likelihood that observed behaviors or lifestyles—whether encountered offline or through digital media—are perceived as legitimate options (Baldwin & Mussweiler, 2018). In contrast, countries characterized by higher normative constraint may attenuate the influence of such cues by elevating the perceived social and psychological costs of deviation, even when exposure is high.

From a broader theoretical perspective, openness to sugar relationships can be understood as a specific manifestation of a more general orientation toward sexual-economic exchange. Across countries and historical periods, intimate relationships have often involved explicit or implicit exchanges of resources, status, security, or material support for intimacy, companionship, or sexual access (Baumeister & Vohs, 2004; Tabet, 2012). Contemporary labels such as “sugar dating” do not introduce a fundamentally new phenomenon; rather, they render visible and name a form of exchange that has long existed in less formalized or less publicly articulated ways. What distinguishes sugar relationships in the digital era is not the presence of exchange per se, but the degree to which such arrangements are openly discussed, symbolically framed, and socially negotiated within online environments (Meskó, 2025).

Importantly, openness to sexual-economic exchange should not be conflated with participation in such relationships. Instead, it reflects an attitudinal disposition—a willingness to consider intimacy involving explicit or asymmetric exchanges of resources as acceptable, imaginable, or personally viable. This distinction is particularly relevant in normatively sensitive domains, where individuals may be exposed to certain relationship models without necessarily endorsing or enacting them. Consistent with broader research on the attitude–behavior gap, openness captures a psychologically meaningful orientation that may precede, but does not imply, behavioral engagement (Ajzen, 1991; Sheeran, 2002). As such, openness to sugar relationships represents a suitable outcome variable for examining how digital exposure and country-level norms jointly shape attitudes toward sexual-economic exchange.

Empirical research further demonstrates that attitudes toward sugar relationships vary substantially across countries. Large-scale cross-country studies reveal pronounced differences in how such arrangements are evaluated, morally framed, and socially legitimized, indicating that openness to sugar relationships is embedded in broader normative environments rather than determined solely by individual-level economic circumstances (Krisch et al., 2019; Meskó et al., 2024). Notably, these differences persist even when accounting for material indicators such as national wealth or gendered economic inequality, reinforcing the view that openness reflects country-level patterns of normative interpretation rather than straightforward responses to structural constraint.

At the same time, digital platforms have played a central role in increasing the visibility and symbolic coherence of sugar relationships. Online environments facilitate not only exposure to sugar-related narratives and lifestyles but also the circulation of peer-generated evaluations concerning attractiveness, success, and social status. In doing so, digital spaces embed sexual-economic exchange within broader repertoires of dating practices and relationship scripts, potentially amplifying its normative salience (Gunnarsson & Strid, 2023; Konings et al., 2022). This heightened visibility may be particularly consequential for younger users, for whom social media constitute primary arenas of social comparison, identity formation, and normative learning.

Despite these developments, existing research has rarely examined how digital exposure and country-level norms are associated with openness to sugar relationships. Prior studies have typically focused either on structural and socioeconomic correlates or on individual-level psychological predictors, often treating digital environments and country context as background conditions rather than interactive forces. As a result, relatively little is known about the conditions under which social media exposure translates into psychologically meaningful openness to sexual-economic exchange.

The present study addresses this gap by integrating individual-level social media use with country-level cultural tightness–looseness in a cross-country multilevel framework. Focusing on young women, we conceptualize openness to sugar relationships as an attitudinal manifestation of broader orientations toward sexual–economic exchange rather than as an indicator of behavioral participation. Using multilevel Bayesian modeling, we test whether the association between social media use and openness to sugar relationships is moderated by cultural tightness–looseness, such that digital exposure is more strongly related to openness in countries characterized by lower cultural tightness.

### Hypotheses

#### **H1**

Higher levels of social media use will be associated with greater openness to sugar relationships among women.

#### **H2**

Cultural tightness–looseness at the country level will moderate the association between social media use and openness to sugar relationships, such that the positive association will be stronger in countries scoring lower on cultural tightness–looseness.

#### **H3**

Cultural tightness–looseness will primarily function as a contextual moderator rather than as a direct predictor of openness to sugar relationships.

#### **H4**

Age will be negatively associated with openness to sugar relationships.

## Method

### Participants

The present study is a secondary analysis of a publicly available cross-country dataset on romantic love and mate preferences collected by Kowal et al. (2025). The original dataset comprises responses from 117,293 participants across 175 countries and was published in *Scientific Data*. The dataset is openly accessible and includes a wide range of demographic, psychological, and relationship-related variables, making it suitable for examining cross-national patterns in attitudes toward intimate and relational phenomena.

For the screening, first, only participants who provided valid responses on measures of openness to sugar relationships, social media use, age, and country of residence were retained. Responses collected from participants who did not provide answers to all items mentioned above were excluded. Also, we removed responses from countries where the country-level data was unavailable. As a result, we extracted responses submitted by 34,318 participants from 33 countries. Then, for the purposes of the present study, analyses were restricted to female participants. Respondents who identified as male or reported a gender other than a woman were excluded. This procedure resulted in the reduction of the sample size to 22,432.

Finally, to ensure a sufficient sample size for the measurement invariance test, measurement alignment, and multilevel modeling, data collected from participants from countries with *n* ≥ 100 were used in the present study. The final analytic sample consisted of 22,292 women from 22 countries. Detailed country-level descriptive statistics for the female analytic sample, including age, relationship status, and employment characteristics, are reported in Supplementary Table S1. All data, analysis scripts, and supplementary materials associated with the present study are openly available via the Open Science Framework (OSF): https://osf.io/whq7j/overview?view_only=d961e5b1de9a446ebf36ceafaefe909d.

### Procedure

The original data were collected via an online, self-report survey administered in multiple languages. All participants provided informed consent prior to participation.

### Measures

#### Individual-Level Measures

##### Openness to Sugar Relationships

Openness to sugar relationships was assessed using the younger women version of the Acceptance of Sugar Relationships in Young Women and Men Scale (ASR-YWMS), previously employed in a large cross-national study by Meskó et al. (2024). The scale measures attitudinal openness toward receiving material or financial resources from an older and wealthier partner in exchange for intimacy or sexual involvement. Responses were recorded on a 5-point Likert-type scale ranging from strongly disagree to strongly agree, with higher scores indicating greater openness. Internal consistency estimates (McDonald’s ω) for all scales across countries are reported in Supplementary Table S2

The present study constitutes a further secondary analysis of the same international dataset used by Meskó et al. (2024), focusing on a distinct research question and analytic framework. Whereas the original study examined global patterns and correlates of attitudes toward sugar relationships, the current analysis specifically investigates the interaction between individual-level social media use and country-level cultural norms. Measurement invariance across countries was examined, and latent factor scores were estimated following alignment procedures to account for cross-country measurement non-invariance.

##### Social Media Use

Social media use was measured with a self-report item assessing the frequency of engagement with social networking platforms. Higher scores indicate more frequent use of social media. This variable was treated as a continuous individual-level predictor in all analyses.

##### Age

Participants reported their age in years. Age was included as a continuous control variable, given prior evidence of age-related differences in mating orientations and openness to sexual-economic exchange.

#### Country-Level Measures

Country-level indicators were merged with individual-level data based on participants’ country of residence. All macro-level variables were treated as contextual characteristics rather than individual attributes.

##### Digital Infrastructure and Accessibility

Internet penetration was operationalized as the percentage of individuals using the internet in each country, based on data from the World Bank (2023a). Smartphone penetration was measured using the number of mobile cellular subscriptions per 100 people, also obtained from the World Bank (2023c). These indicators capture the extent to which digital infrastructure enables exposure to and dissemination of social media-based relationship scripts and lifestyles.

##### Cultural Tightness–Looseness

Cultural tightness–looseness was measured using country-level indices developed by Uz (2014), including domain-specific cultural tightness–looseness (CTL_DS), domain-general cultural tightness–looseness (CTL_DG), and a combined index (CTL_C). Higher values indicate looser normative environments characterized by greater tolerance for behavioral deviation. Consistent with the theoretical framework, cultural tightness–looseness was treated primarily as a contextual moderator rather than as a direct predictor of individual attitudes.

##### Socioeconomic Structure

Socioeconomic structural conditions were captured using gender-related indicators from the World Bank. Gender wage gap-related indicators were derived from female labor force participation statistics (World Bank, 2023b). Female youth unemployment was operationalized as the percentage of unemployed females aged 15–24 (World Bank, 2023d). These variables reflect structural constraints and incentives that may shape the broader context of sexual–economic exchange.

##### Media and Internet Freedom

The openness of the online information environment was assessed using Freedom on the Net country scores published by Freedom House (2024). This index captures constraints on internet access, content restrictions, and violations of user rights. Higher scores indicate more open and independent online information environments.

### Analytic Strategy

#### Measurement Invariance Test and Measurement Alignment

Given the data were collected from different countries, we first examined the measurement invariance of the ASR-YWMS (Meskó et al., 2024). This procedure was performed to ensure that the measurement model of the ASR-YWMS was equally valid across different groups (Fischer & Karl, 2019). It is necessary to confirm that the latent factor of our interest, openness to sugar relationships, was not measured and quantified across countries in a biased manner.

We tested the measurement invariance of the ASR-YWMS via multigroup confirmatory factor analysis (MG-CFA) (Putnick & Bornstein, 2016). The measurement model to be confirmed was created based on the original model established and validated by Meskó et al. (2024). We conducted CFA with different model constraints while setting the country as a group variable. The different levels of measurement invariance that we tested follow standard definitions (configural, metric, scalar, and residual invariance; Putnick & Bornstein, 2016). Whether each level of invariance was achieved could be confirmed by reviewing model fit indicators, i.e., RMSEA, SRMR, CFI, and TLI (Han, 2024a). In the case of configural invariance, the indicators were supposed to demonstrate acceptable model fit, e.g., RMSEA and SRMR < .08, and CFI and TLI ≥ .90 (Hu & Bentler, 1999). For the rest of the invariance levels, changes in the indicators (i.e., the differences in the indicators before versus after applying additional model constraints) were examined. The criteria for metric invariance were ΔRMSEA and ΔSRMR <  + .30, and ΔCFI and ΔTLI ≥ − .01, and for scalar and residual invariance, ΔRMSEA and ΔSRMR <  + .15, and ΔCFI and ΔTLI ≥ − .01 (Chen, 2007). Previous psychometrics research suggests that at least metric invariance should be established to perform regression analysis and scalar invariance is required to compare between-group mean differences appropriately (Han, 2024a; Putnick & Bornstein, 2016). Accordingly, we also tested whether scalar invariance was supported by our data before conducting further analyses to test our hypotheses.

Although metric, not scalar, invariance is required for regression analysis—the main analysis method employed in the present study (Lee et al., 2016)—we decided to aim for scalar invariance because we will report the mean scores and standard deviations of the construct by countries for readers’ information, and such may potentially be used for between-group comparison. We conducted the hypothesis testing again with the latent factor scores calculated with a measurement model based on the metric invariance assumption to assure that the employment of the stricter invariance criterion would not significantly alter the result.

If the minimally required degree of measurement invariance, scalar invariance, was not achieved, measurement alignment was performed to address the non-invariance across groups. The measurement alignment is a process to adjust group means and intercepts, and accordingly, factor loadings and intercepts to minimize inter-group biases automatically (Asparouhov & Muthén, 2014; Han, 2024a). Once the procedure is successfully done, existing non-invariance is supposed to be absorbed, so further statistical analysis and group mean comparison become possible. To evaluate whether the non-invariance issue was satisfactorily resolved, several measurement alignment indicators should be examined. First, *R*^*2*^_loadings_ and *R*^*2*^_intercepts_, which report the extent to which non-invariance existing across factor loadings and intercepts was absorbed, respectively, should be .75 or higher (Fischer & Karl, 2019; Rachev et al., 2021). Second, when the item-level invariance was tested, no more than 25% of the items should demonstrate significant deviations both in terms of factor loadings and intercepts (Asparouhov & Muthén, 2014). Finally, a Monte Carlo simulation should be performed to test whether the group means calculated by CFA with the scalar invariance assumption (true means) and those by alignment (estimated means) were strongly correlated with each other. The resultant rank-order correlation coefficient should be .95 or higher (Blackburn et al., 2023; Muthén & Asparouhov, 2018).

Following the guidelines overviewed above, we first tested measurement invariance via MG-CFA with the *lavaan* package in R (Rosseel, 2012). Because responses were anchored to an ordinal scale, we employed the WLSMV estimator, which is deemed to be suitable for CFA with ordinal observed variables (Li, 2016). We conducted MG-CFA with four levels of model constraints (configural, metric, scalar, and residual invariance). We confirmed whether a specific level of invariance was supported by the data by examining the changes in model fit indicators, i.e., RMSEA, SRMR, CFI, and TLI, as described above.

If scalar invariance was not achieved, we performed measurement alignment with the *sirt* package in R (Robitzsch, 2025a). We first reviewed measurement alignment indicators to evaluate whether the alignment process successfully addressed existing measurement non-invariance. Estimation parameter options were determined based on recommendations made by Han (2024a) and Robitzsch (2025b, 2025c). We examined the resultant measurement alignment indicators, i.e., *R*^*2*^_loadings_, *R*^*2*^_intercepts_, and the proportions of items demonstrating significant deviations in factor loadings and intercepts. Furthermore, Monte Carlo simulations were performed to examine whether the true and estimated group means were sufficiently correlated with each other. The simulations were conducted with a code provided by Han (2024a) for *n* = 100, 200, and 500. For each condition, simulations were done for 100 times via multiprocessing implemented in BayesFactorFMRI (Han, 2021). We used the averaged correlation coefficients for each condition for quality assessment.

Once we confirmed that the non-invariance issue was successfully resolved, we calculated the latent factor score of the ASR-YWMS with the aligned factor loadings and intercepts for further analyses. We employed the adjusted latent factor score estimation method proposed by Han (2024a).

Given the lack of scalar invariance, alignment was used to obtain comparable latent scores across countries while minimizing bias due to measurement non-invariance. This approach allows for cross-national comparisons of latent constructs even when full scalar invariance is not achieved.

#### Bayesian Multilevel Modeling

To account for the hierarchical structure of the data, with individuals nested within countries, analyses were conducted using multilevel modeling. Bayesian multilevel models were estimated to examine associations between individual-level social media use and openness to sugar relationships, as well as the moderating role of country-level cultural tightness–looseness. Random intercepts were specified at the country level. The Bayesian method was employed instead of the conventional frequentist method as the Bayesian approach allows researchers to directly test hypotheses of interest (e.g., alternative hypotheses) instead of to test whether null hypotheses should be rejected (Han, 2024b). Furthermore, it is useful to examine the extent to which each individual model is strongly supported by evidence compared with others (Wagenmakers et al., 2018). The capacity for model comparison is beneficial in our study because we intend to compare multiple models with different predictors included (e.g., main effects only vs. with an interaction effect) to test our hypotheses (Blackburn et al., 2024).

Model comparison was performed using Bayes Factors (BF) to evaluate the relative fit of competing models with differing combinations of predictors and covariates. A model BF quantified the extent to which a specific model of interest was more strongly supported by evidence compared with another model (Wagenmakers et al., 2018). In this study, we calculated model BFs comparing candidate models versus a null model without any predictors included. We deemed that a model of interest was significantly better than another when the calculated 2log BF comparing the likelihood of the two models was 2.00 or higher (Kass & Raftery, 1995). For our hypothesis testing, three models were compared:

Model 0 (null model): only includes countries as a random effect

Model 1 (main effect-only model): Model 0 + social media use and CTL_C as main effects + age for the optional test

Model 2 (interaction effect model): Model 1 + the interaction effect of social media use x CTL_C

In addition to these models, for exploration, we also tested another model including covariates:

Model 3: Model 2 + macroscopic-level covariates (i.e., digital infrastructure and accessibility, socioeconomic structure, media and internet freedom)

We calculated model BFs for main hypothesis testing, i.e., BF10 (Model 1 vs. Model 0) and BF20 (Model 2 vs. Model 1). Once the best model showing the highest model BF value was identified, we examined whether the included predictors were statistically significant with the BF of each predictor. Like model BFs, we concluded that a hypothesis regarding a specific predictor was supported when the resultant 2log BF of the predictor was 2.00 or higher. Finally, we compared Model 3, which included covariates, and the identified best model (either Model 2, 1, or 0) to explore whether the addition of macroscopic-level covariates was warranted by the data. The BF of each covariate was also examined for exploration.

H1 was tested by whether the BF of the predictor of interest, social media use, was higher than the cutoff value. If none of Model 1 or 2, which included social media use as a predictor, was found to be superior to Model 0, i.e., the null model, then H1 would be automatically rejected. H2 and H3 were first examined by whether Model 2 was significantly better supported by evidence compared with Models 0 and 1. Then, we examined the BF of the interaction effect of social media use x CTL_C (2 logBF ≥ 2.00) to confirm H2. H3 was tested by examining whether the 2log BF of the main effect of CTL_C was lower than 2.00. Finally, the optional hypothesis test regarding age examined whether the predictor BF of age was higher than the cutoff, 2log BF ≥ 2.00, in the identified best model. Furthermore, we also explored the significance of the macroscopic-level covariates by comparing Model 3 with the previously identified best model. We assumed that the inclusion of the macroscopic-level covariates would significantly contribute to improving the prediction of the ASR-YWMS with the model BF, 2log BF ≥ 2.00.

Bayesian multilevel modeling was conducted with the R package, *brms* (Bürkner, 2017). All candidate models were estimated with the package, and the resultant model BFs and predictor BFs were tested thereafter. For prior distributions, we used weakly informative Cauchy (0, 1) priors for regression coefficients. This choice reflects the expectation that effects are centered around zero but allows for moderate effect sizes when supported by the data, while providing regularization to prevent overfitting. Such priors are commonly recommended for Bayesian regression models and model comparison in psychological research (Rouder & Morey, 2012; Wagenmakers et al., 2018). During the process, all variables were standardized with the grand mean for better convergence and interpretation of effect sizes.

For sensitivity check, we examined whether the findings, particularly the BFs of main and interaction effects of interest tied to our hypotheses, would not significantly alter when different prior distributions were applied. It has been widely employed for robustness check in quantitative analyses using Bayesian methods (e.g., Han & Park, 2019). This measure was employed in our study to assure that the findings were robust so that they did not rely on the selection of the default Cauchy prior distribution. Following prior literature regarding Cauchy prior distribution selection, we utilized the ultrawide, narrow, ultranarrow, and extremely narrow distributions (Rouder & Morey, 2012; Rouder et al., 2016; Wagenmakers et al., 2018), which entail all practically plausible cases.

## Results

### Measurement Invariance Test and Measurement Alignment

First, we conducted MG-CFA to test the measurement invariance of the ASR-YWMS. When configural invariance was tested, model fit indicators suggested a good fit, RMSEA = .043, SRMR = .010, CFI = .995, TLI = .990, so this level of invariance was supported. Metric invariance was also established as the changes in the fit indicators were: RMSEA = .059, SRMR = .031, CFI = .983, TLI = .981, ΔRMSEA =  + .015, ΔSRMR =  + .020, ΔCFI = − .012, ΔTLI = − .009. However, the minimum requirement for analyses, scalar invariance, was not supported, RMSEA = .107, SRMR = .056, CFI = .919, TLI = .936, ΔRMSEA =  + .048, ΔSRMR =  + .025, ΔCFI = − .064, ΔTLI = − .045. Hence, we decided to conduct measurement alignment and calculate the latent factor score of the ASR-YWMS with the adjusted factor loadings and intercepts for further analyses.

All indicators to evaluate whether the alignment process was successful were found to be satisfactory, so we concluded that measurement alignment was able to address the non-invariance issue in the ASR-YWMS. The first set of indicators, i.e., *R*^*2*^_loadings_ and *R*^*2*^_intercepts_, were .9963 (99.63%) and .9964 (99.64%), respectively. Both were higher than the threshold value, i.e., .75. When the item-level deviations were examined, no item reported significant deviations in factor loadings while 21.8% showed significant deviations in intercepts (see the supplementary *R* output file for the full results at https://osf.io/whq7j/files/92px3?view_only=d961e5b1de9a446ebf36ceafaefe909d). Those were below the cutoff, 25%. Finally, Monte Carlo simulations also reported acceptable results. For all conditions, *n* = 100, 200, and 500, the resultant averaged correlation coefficients about the correlation between the true and estimated group means were strong, .98 (SD = .04), .99 (SD = .02), and .99 (SD = .01), respectively. In all cases, the coefficients were higher than the threshold, .95.

Given that all alignment quality indicators were satisfactory, we concluded that the alignment process was successfully done and the non-invariance issue in the ASR-YWMS was satisfactorily addressed. For planned Bayesian multilevel modeling and hypothesis testing, we calculated the latent factor scores of the ASR-YWMS with the adjusted factor loadings and intercepts across countries. For further information, the descriptive statistics of variables of interest, including ASR-YWMS estimated with the adjusted parameters, are presented in Tables S3.A (individual-level variables) and S3.B (country-level variables including covariates for exploration).

### Bayesian Multilevel Modeling

For main hypothesis testing, we conducted Bayesian multilevel modeling and compared the proposed candidate models, Models 0, 1, and 2. The result of model comparison was that Model 2, which included all main effects of interest, i.e., social media use and CTL_C, the interaction effect of social media use x CTL_C, and age, was best supported by the data. The resultant model BF comparing Model 1 versus Model 0, 2log BF10, was 420.98, and that comparing Model 2 versus Model 0, 2log BF20, was 427.67. The estimated regression coefficient, standard error, predictor BF, and 95% Bayesian credible interval of each predictor of interest are reported in Table 1. The *R* output file containing the full model estimation results for Models 0, 1, 2, and 3 is available at https://osf.io/whq7j/files/58jt3?view_only=d961e5b1de9a446ebf36ceafaefe909d.

**Table 1:** Bayesian multilevel modeling result with Model 2

Predictor	*Β*	*SE*	2log BF	95% CI
Intercept	.07	.06	–	[− .05, .19]
Age	− .11	.01	Infinite	[− .12, − .10]
Social media use	.14	.01	73.66	[.13, .15]
Cultural tightness–looseness	− .02	.06	− 3.17	[− .14, .10]
Interaction effect	.04	.01	55.85	[.02, .05]

**Fig. 1:**
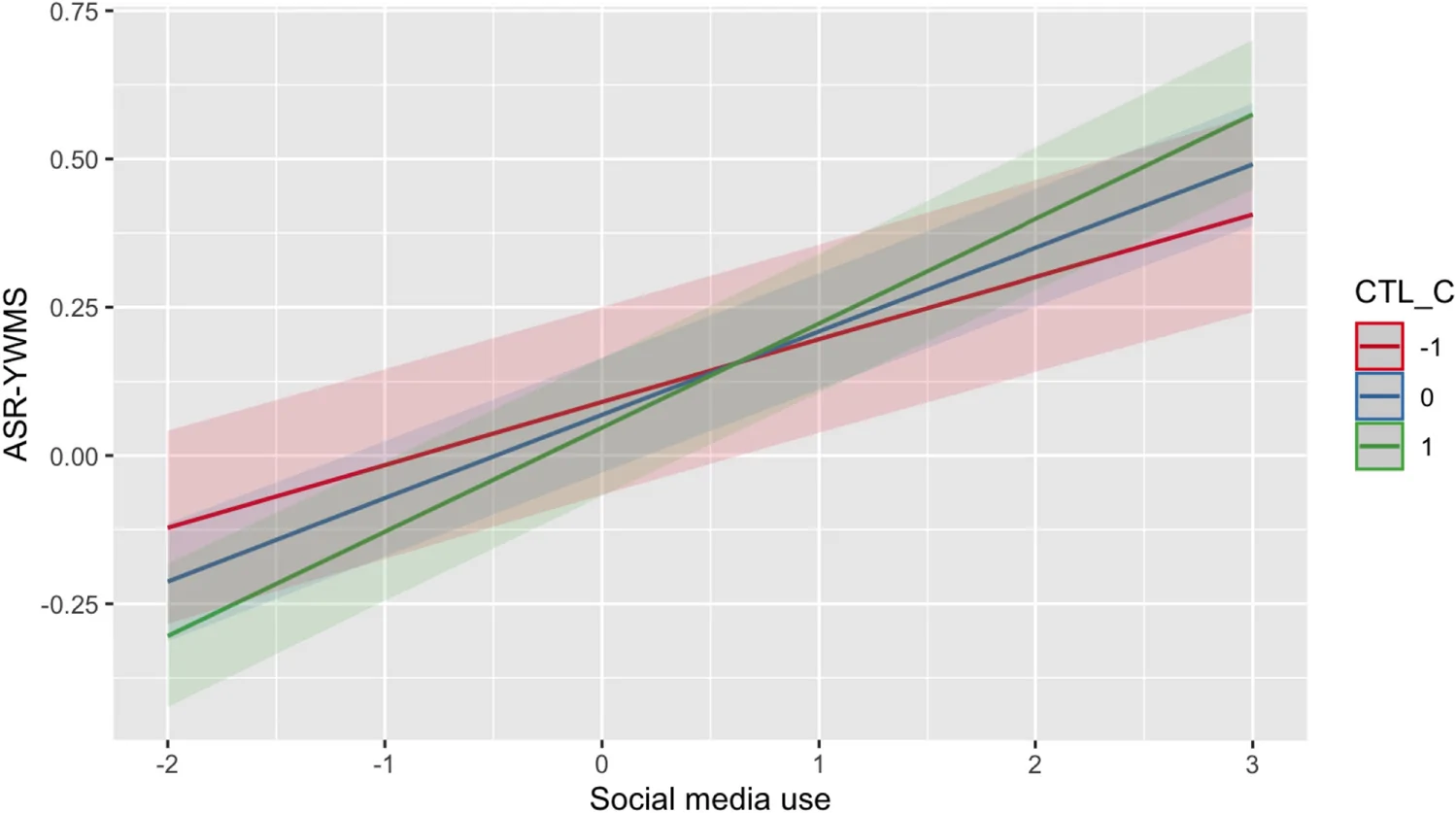
Visualization of the interaction effect of social media use x cultural tightness–looseness on openness to sugar relationships. *Note*: Colored figures available in electronic version only

These results propose that all our main hypotheses were supported. H1 was supported because the 2log BF of the main effect of social media use was 73.66, which was significantly higher than the 2.00 cutoff, and its standardized coefficient was positive. H2 was also accepted as 2log BF of the interaction effect of social media use x CTL_C was 55.85 and its sign was positive (see Fig. 1 visualizing the interaction effect). Finally, the non-significant main effect of CTL_C (2log BF = − 3.17) along with the significant interaction effect examined above supported H3. The optional hypothesis, E1, was also confirmed because the 2log BF of age was infinite and its sign was negative. However, the addition of macroscopic-level covariates (i.e., digital infrastructure and accessibility, socioeconomic structure, media and internet freedom) was not justified as the model BF of Model 3, 2log BF30, was 416.22; it was significantly lower than 2log BF20, the model BF of the previously identified best model, Model 2.

### Sensitivity Check

First, we tested our hypotheses with latent scores estimated based on the metric invariance assumption to assure that the employment of the stricter assumption, scalar invariance, would not significantly alter the findings. The model BFs showed that Model 2 with the interaction effect was best supported by the data, 2log BF10 = 758.60; 2log BF20 = 767.21; and 2log BF30 = 732.82, identical to the original findings. All effects of interest, the main effect of social media use, and the interaction effect between social media use and CTL_C were statistically significant. In conclusion, the hypotheses of interest still held when the metric invariance requirement was applied in lieu of the scalar invariance requirement.

Second, as a part of sensitivity check, we examined whether the findings would significantly alter when different Cauchy scales were applied (Han & Park, 2019; Rouder & Morey, 2012; Rouder et al., 2016; Wagenmakers et al., 2018). In all cases when the prior distribution was ultrawide (Cauchy [0, 1.41]), narrow (Cauchy [0, .71]), ultranarrow (Cauchy [0, .50]), and extremely narrow (Cauchy [0, .20]), the main and interaction effects of interest remained statistically significant. 2log BFs of the main effect of social media use were 116.37, Infinite, Infinite, and Infinite when the ultrawide, narrow, ultranarrow, and extremely narrow Cauchy priors were applied. Similarly, the resultant 2log BFs of the interaction effect were 71.84, Infinite, Infinite, and Infinite, respectively. In conclusion, the results of the hypothesis tests did not significantly change even with changes in the prior distributions.

### Exploratory Analyses

#### Leave-One-Out Analysis at the Country Level

In addition to the planned hypothesis tests, we also conducted several exploratory analyses for additional information. First, given that we found the significant interaction effect between social media use and CTL_C, we examined whether such a significant outcome would be attributable to possible outliers at the country level. We performed a series of leave-one-out analysis and examined whether the predictors of interest were still statistically significant in terms of 2log BFs even after excluding one specific country at a time. As shown in a supplementary table available at https://osf.io/whq7j/files/b4smz?view_only=d961e5b1de9a446ebf36ceafaefe909d, in all 22 cases, both the main effect of social media use and the interaction effect between social media use and CTL_C were significant (2log BF ≥ 2.00). The results suggest that the original findings were not altered even when data from one specific country was excluded, so the significant main and interaction effects of interest might not be solely attributable to possible outliers at the country level.

#### Consideration of Individual-Level Socioeconomic Status

Second, we examined whether the inclusion of individual-level socioeconomic status in the model would significantly alter the estimation result. We examined the model including macroscopic-level socioeconomic structure-related variables, Model 3; however, while conducting planned hypothesis tests, individual-level socioeconomic status was not considered. Thus, we added the individual-level socioeconomic status to Model 2 and investigated whether its inclusion would significantly influence the hypothesis testing results.

Even when the additional factor was added to the model, the main effect of interest, social media use, *β* = .14, *SE* = .01, 95% CI = [.13, .15], 2log BF = Infinite, and the interaction effect between social media use and CTL_C were still statistically significant, *β* = .03, *SE* = .01, 95% CI = [.02, .05], 2log BF = Infinite. Interestingly, the added socioeconomic status at the individual level negatively predicted the dependent variable of interest, *β* = -.03, *SE* = .01, 95% CI = [-.04, -.01], 2log BF = 4.08.

#### Bayesian Multilevel Modeling with Random Slopes

Third, we also examined whether the relationship between the predictors of interest and the dependent variable varied across countries by adding random slopes to the best model, Model 2. For this analysis, we freed the slope of social media use at the individual level across countries. When we compared the new model with random slopes to Model 2, the new model was significantly better than Model 2, 2log BF = 153.04. However, the main effect of social media use, *β* = .13, *SE* = .02, 95% CI = [.11, .16], 2log BF = Infinite, and the interaction effect, *β* = .04, *SE* = .02, 95% CI = [.01, .07], 2log BF = 9.45, remained statistically significant, so the overall findings were not significantly altered by the addition of random slopes. We created a supplementary plot showing the different estimated slopes pertaining to the relationship between social media use and ASR-YWMS across 22 different countries; the plot is available at https://osf.io/whq7j/files/6xny2?view_only=d961e5b1de9a446ebf36ceafaefe909d.

## Discussion

### Summary of Main Findings

The present study examined how social media use and cultural tightness–looseness jointly shape openness to sugar relationships among women across countries. The results indicate that social media use is positively associated with openness to sugar relationships and that this association is modestly stronger in culturally looser countries. Cultural tightness–looseness did not emerge as a direct predictor of openness but showed a moderating effect on the relationship between digital exposure and attitudes toward sugar relationships. In addition, age showed a robust negative association with openness, indicating that receptivity to sugar relationships is lower at higher ages.

By integrating individual-level attitudes with country-level normative indicators across one of the largest cross-country datasets currently available, the present study moves beyond country-specific or regionally bounded accounts of sugar relationships. Instead, it provides a global test of how digital exposure and cultural normativity jointly shape openness to sexual-economic exchange.

The additional sensitivity and exploratory analyses further supported the stability of the observed interaction pattern. The association between social media use and openness to sugar relationships remained detectable across different measurement invariance assumptions, alternative prior specifications, and exploratory model extensions, including random slope models and models controlling for individual-level socioeconomic status. Although the interaction effect size remained modest, these findings suggest that the observed association was not reducible to a single modeling decision, prior specification, or influential country-level configuration. Rather, the interaction appeared to reflect a relatively stable—albeit small—cross-national pattern linking digital exposure and normative permissiveness.

### Cultural Tightness–Looseness as a Normative Gatekeeper of Digital Sexual Socialization

The findings suggest that cultural tightness–looseness operates not as an independent driver of openness to sugar relationships but as a contextual condition that is associated with whether social media exposure is psychologically consequential. In countries characterized by lower cultural tightness, where norms are less strictly enforced and behavioral diversity is more readily tolerated, exposure to sugar-related content and lifestyles through social media appears more likely to translate into attitudinal openness. In contrast, countries scoring higher on cultural tightness may be associated with a weaker association even when exposure is present.

This pattern aligns with theoretical accounts conceptualizing cultural tightness–looseness as a dimension governing norm strength, tolerance for deviance, and behavioral regulation (Gelfand et al., 2011; Uz, 2014). Importantly, the absence of a main effect of cultural tightness–looseness underscores that normative permissiveness alone does not generate openness to sugar relationships. Rather, permissiveness appears to be associated with the internalization of alternative relationship scripts encountered in digital environments. This interpretation is consistent with research showing that country-level normative contexts shape the meaning and acceptability of social comparisons and emerging norms, rather than directly producing specific attitudes or behaviors (Baldwin & Mussweiler, 2018).

Taken together, these findings suggest that cultural tightness–looseness should be understood not merely as a distal background characteristic but as one of the psychological filters through which globally circulating sexual scripts may acquire—or fail to acquire—attitudinal relevance at the individual level.

### Social Media Use as Conditional Sexual Socialization

A substantial body of research has demonstrated that exposure to sexualized media content plays an important role in shaping sexual attitudes, perceived peer norms, and expectations about intimate relationships (Brown et al., 2006; Coyne et al., 2019; Ward, 2003). Social media platforms extend these processes by combining sexualized content with peer comparison, algorithmic amplification, and lifestyle-based self-presentation. As a result, they constitute a particularly potent environment for the dissemination of relationship models that blend intimacy, status, and material resources.

However, the present findings indicate that social media use does not exert a uniform influence on openness to sugar relationships across countries at different levels of cultural tightness–looseness. Rather than functioning as a universal risk or exposure factor, social media appears to act as a conditional agent of sexual socialization, whose psychological impact depends on the surrounding normative climate. In countries characterized by lower cultural tightness digitally transmitted sexual-economic narratives may be somewhat more likely to be legitimized and cognitively integrated, whereas in countries characterized by higher cultural tightness, stronger normative constraints may limit their attitudinal uptake. This pattern is consistent with social influence models suggesting that informational exposure translates into preferences primarily when social constraints are weak, ambiguous, or contested (Jones & DuVal, 2019).

Importantly, these findings highlight that exposure alone is insufficient for understanding cross-national variation in sexual-economic attitudes at a global level. While the present study focused on overall social media use, it is important to recognize that not all forms of exposure are equivalent. Certain types of content—particularly highly curated, aspirational, or “glamorized” lifestyle portrayals—may disproportionately contribute to the normalization of sugar relationships by framing them as desirable or attainable. Future research could benefit from distinguishing between general social media engagement and exposure to such specific narratives.

### Openness to Sugar Relationships as a Manifestation of Sexual–Economic Exchange Orientation

The present analyses focus exclusively on women, reflecting the structurally gender-asymmetric nature of sexual-economic exchange. In most empirical contexts, including sugar relationships, men are more likely to occupy the role of resource providers, whereas women are more likely to be positioned as recipients of material benefits in exchange for intimacy or sexual availability. As a result, openness to such arrangements reflects conceptually distinct processes in women compared to men. Prior research has shown that openness to sugar relationships in women is associated with specific psychological and emotional vulnerabilities, as well as distinct relational motivations (Meskó et al., 2026b), while men’s attitudes toward such arrangements follow different patterns and motivational structures (Meskó et al., 2024). Accordingly, including men within the same analytical framework would introduce qualitatively different processes, potentially obscuring the mechanisms under investigation.

The results of the present study support a conceptualization of openness to sugar relationships as a specific manifestation of a broader sexual-economic exchange orientation, rather than as an isolated or deviant attitude. Previous research has shown that openness to sugar relationships is more strongly associated with short-term mating orientation than with life history strategy or structural deprivation, suggesting that it reflects a psychologically coherent orientation toward intimacy and exchange (Meskó et al., 2024, 2026b).

Building on this framework, Orientation toward Sexual-Economic Exchange has been proposed as a higher-order psychological dimension capturing individual differences in how sexual intimacy is cognitively linked to material resources, status, and instrumental outcomes (Meskó, 2026). From this perspective, openness to sugar relationships reflects one contemporary instantiation of this orientation, shaped by digital visibility and culturally specific norms. Viewed in this way, openness to sugar relationships represents not an anomalous or marginal attitude but a psychologically intelligible response to contemporary configurations of intimacy, material resources, and digital visibility.

The present findings reinforce this interpretation by demonstrating that openness appears to be shaped in part by the interaction between digital exposure and normative permissiveness, rather than by infrastructural access or economic constraints alone.

### The Limited Role of Structural and Infrastructural Factors

Contrary to intuitive expectations, the inclusion of macro-level indicators related to digital infrastructure, socioeconomic structure, and internet freedom did not substantially improve model fit beyond the interaction between social media use and cultural tightness–looseness. Although these factors may influence exposure opportunities or background incentives, they did not constitute primary explanatory mechanisms for openness to sugar relationships in the present analyses.

From a Bayesian perspective, the preference for the more parsimonious model suggests that the additional explanatory power offered by these covariates was insufficient to justify increased model complexity. Conceptually, this pattern highlights the importance of distinguishing between structural access and psychological meaning. While access to digital platforms may be necessary for encountering sugar-related narratives, it does not determine whether such narratives are interpreted as legitimate, desirable, or personally relevant. Similarly, socioeconomic constraints may shape the perceived utility of transactional intimacy but do not appear sufficient to explain openness in the absence of supportive normative contexts.

This distinction is particularly important when interpreting cross-national variation in digitally mediated forms of intimacy.

### Age and Life-Course Considerations

Age emerged as a consistent negative predictor of openness to sugar relationships, even after controlling for social media use and cultural context. This finding is consistent with prior research indicating that openness to sugar relationships is more pronounced among younger women and declines across early adulthood (Meskó et al., 2024). Rather than reflecting a stable personality disposition, openness appears to be developmentally situated, aligning with life-course perspectives that emphasize changing mating priorities, risk tolerance, and identity exploration.

Importantly, the persistence of the interaction effect after controlling for age indicates that the observed cultural and digital dynamics are not fully accounted for by age-related differences. However, given the cross sectional nature of the data, it is not possible to disentangle age, cohort, and period effects. Instead, they reflect broader processes of sexual socialization that operate most strongly during periods of heightened relational experimentation.

One possible interpretation of the observed age pattern relates to generational differences in digital immersion. Younger individuals are more likely to have developed their relational expectations within online environments where such scripts are more visible and socially normalized. However, given the cross sectional nature of the data, this interpretation should be considered tentative and cannot be disentangled from age-related effects.

### Limitations and Future Directions

Several limitations should be acknowledged. First, the cross sectional nature of the data precludes causal inference regarding the directionality of the observed associations. Second, the focus on attitudinal openness does not allow direct conclusions about engagement in sugar relationships or other forms of transactional intimacy. Third, cultural indicators were aggregated at the country level and may not fully capture within-country variation in normative climates.

Future research could address these limitations by employing experimental designs that manipulate exposure to sugar-related content, longitudinal approaches that track attitudinal change over time, or multilevel assessments incorporating perceived local norms. Integrating behavioral and neuroscientific methods may further clarify the motivational and cognitive mechanisms underlying sexual–economic exchange orientations.

In addition, the present study did not include several theoretically relevant individual-level mechanisms that may shape how social media use relates to openness to sugar relationships. In particular, prior work suggests that the internalization of material values, the objectification of the body, and different patterns of social media engagement (including intensive or problematic use) may play a role in how digitally transmitted relationship scripts are interpreted and incorporated into individual attitudes. These processes likely operate alongside the broader cultural and structural conditions examined here. However, the present design prioritized a macro-level, cross-national perspective, and therefore did not capture these more proximal psychological pathways. Future research integrating detailed individual-level measures with cross-cultural designs would allow for a more fine-grained understanding of how digital exposure and normative environments jointly shape sexual-economic exchange orientations.

### Implications

The present findings have several theoretical and practical implications. Theoretically, they support a multilevel perspective on sexual attitudes by demonstrating that digitally mediated exposure does not operate in isolation but is filtered through culturally embedded normative frameworks. This highlights the importance of integrating individual-level media effects with macro-level cultural dimensions when examining emerging forms of intimacy. Practically, the results suggest that interventions or public discourse addressing transactional relationships should consider the broader normative environment in which such attitudes develop. Efforts focusing solely on digital exposure may be insufficient if they do not account for the cultural conditions that enable or constrain the internalization of such relationship scripts.

### Conclusions

Taken together, the present findings demonstrate that openness to sugar relationships appears to be shaped not only by digital exposure or structural conditions alone but also by their interaction with country-level normative frameworks. Understanding this interaction may help explain why digital environments are associated with differences in intimate orientations across countries, although the present study does not directly assess equivalence in exposure or access conditions. By situating openness within a broader sexual-economic exchange orientation, this study contributes to a more integrated psychological understanding of transactional intimacy in the digital age.

## Supplementary Information

Below is the link to the electronic supplementary material.

Supplementary file1 (DOCX 38 KB)

## Data Availability

All data and Supplementary Materials have been made publicly available at the Open Science Framework (OSF) and can be accessed at https://osf.io/whq7j/overview?view_only=d961e5b1de9a446ebf36ceafaefe909d.
