# Supplementary material for: Social Media Use and Openness to “Sugar Relationships”: Cultural Tightness–Looseness as a Moderator Across 22 Countries
Source: Arch Sex Behav. 2026 Oct 22;55(7):3097–109. doi: 10.1007/s10508-026-03551-w (PMC13748100; doi:10.1007/s10508-026-03551-w)
Supplement: Supplementary file 1 [file 10508_2026_3551_MOESM1_ESM.docx]

**Social Media Use and Openness to “Sugar Relationships”: Cultural Tightness–Looseness as a Moderator Across 22 Countries**

**Supplementary Materials**

[Instruction for the translators when translating the survey into local languages	2](#_Toc126406967)

[Data collection	3](#_Toc126406968)

[Table S1.A. Detailed participants’ descriptive characteristics across countries (age, gender, sex at birth).	4](#_Toc126406969)

[Table S1.B. Detailed participants’ descriptive characteristics across countries (relationship status, employment).	8](#_Toc126406970)

[Table S2. Descriptive statistics along languages.	12](#_Toc126406971)

[Table S3. Reliability coefficients (McDonald's ω) of scales used in the study along languages.	13](#_Toc126406972)

Instruction for the translators when translating the survey into local languages

How should the translation look?

We follow the guidelines of WHO (https://www.who.int/substance_abuse/research_tools/translation/en/). In brief:

1. At least one (ideally more than one) person will do a forward translation (from English to your native language). If more than one person is involved in this part, they work together and discuss all issues. Once this is done, it is time for:

2. At least one (ideally more than one) person will now take the version of the questionnaire translated by the other team and translate this version back to English from your native language. If more than one person is involved in this part, they work together and discuss all issues. Once this is done, it is time for:

3. At the end, all translators compare two versions of the questionnaire and spot any differences. Please, at this point, try to figure out why such differences appeared. Do they substantially change the meaning? Maybe some questions would need slight alterations? Our goal is to investigate psychological phenomena. Thus, the equivalence of our measures is super important. If we do not achieve the equivalence of invariance, we would not draw any conclusions from our study, as any potential differences between our participants from different countries would be attributed to the differences in measures and not underlying across individuals or countries factors.

4. Please, copy the final version into the sheet called “FINAL” and let me know. I will then upload your translation into Qualtrics and send you the link to the study. I will then ask you to make sure that everything is ok and nothing got lost or twisted in the process.

5. IMPORTANT THINGS:

During the translation, one of the most essential things would be to translate the questions/items but WITHOUT changing anything else. I know that the excel file looks a bit messy, but that is because it has some HTML formatting inserted (so to make our survey look more visually appealing). If you, by accident, delete some “<” signs, it will result in errors in the layout of the study. So please, translate ONLY English words and ignore the rest, leaving it unaltered (as shown in the excel file, sheet “INSTRUCTIONS”).

If the local language in your country is SIMILAR (or identical) to languages of other countries (e.g., Spanish, Portuguese), please, let me know. I can then assign you to the given language translation group (so you would join forces).

All the best,
Marta Kowal

Data collection

Data were collected mostly online in all but two countries*,* as participants from Algeria and Morocco could not access the Qualtrics website. Thus, collaborators collected data in person using a paper-pencil method. Furthermore, Iranian participants also had difficulties accessing the Qualtrics website, so we re-created the survey and collected data using Google Forms. One Russian Collaborator collected data using the Toloka website (a crowdsourcing platform popular in Russia)*.*

Table S1. Detailed participants’ descriptive characteristics across countries included in the analyzed dataset after screening (age, employment status).

| Country | *n* | Age | | Relationship status | | | |
| --- | --- | --- | --- | --- | --- | --- | --- |
|  |  | Mean | *SD* | Single (%) | Dating (%) | In a committed | Married (%) |
|  |  |  |  |  |  | relationship (%) |  |
| All | 22,292 | 28.96 | 11.65 | 40.22% | 13.02% | 27.13% | 19.63% |
| Argentina | 483 | 39.81 | 16.11 | 56.25% | 13.54% | 5.63% | 24.58% |
| Canada | 381 | 32.09 | 10.60 | 34.91% | 9.45% | 29.66% | 25.98% |
| Chile | 921 | 29.66 | 7.17 | 37.28% | 30.22% | 19.67% | 12.83% |
| Estonia | 303 | 33.27 | 12.19 | 37.95% | 16.17% | 25.74% | 20.13% |
| France | 1,155 | 27.28 | 10.94 | 42.08% | 10.04% | 39.05% | 8.83% |
| Germany | 372 | 27.94 | 9.37 | 37.47% | 6.20% | 36.12% | 20.22% |
| Hungary | 414 | 25.71 | 9.13 | 29.95% | 6.04% | 50.24% | 13.77% |
| Iran | 336 | 29.90 | 8.48 | 40.77% | 16.96% | 6.85% | 35.42% |
| Italy | 2,127 | 30.85 | 12.22 | 30.10% | 8.56% | 48.87% | 12.46% |
| Japan | 880 | 38.58 | 12.00 | 39.89% | 14.09% | 0.80% | 45.23% |
| Mexico | 159 | 29.28 | 8.41 | 35.22% | 32.08% | 9.43% | 23.27% |
| Morocco | 1,078 | 29.37 | 5.91 | 26.44% | 25.88% | 22.63% | 25.05% |
| Netherlands | 269 | 41.80 | 16.86 | 30.11% | 4.09% | 26.77% | 39.03% |
| Nigeria | 309 | 21.79 | 4.65 | 65.37% | 18.77% | 9.06% | 6.80% |
| Philippines | 1,168 | 25.05 | 9.33 | 53.25% | 11.39% | 25.43% | 9.93% |
| Russia | 2,004 | 25.60 | 9.55 | 39.32% | 14.52% | 22.36% | 23.80% |
| Serbia | 938 | 26.32 | 9.44 | 44.56% | 13.65% | 28.78% | 13.01% |
| Turkey | 5,087 | 26.18 | 10.34 | 45.80% | 10.19% | 29.85% | 14.16% |
| Uganda | 144 | 26.30 | 7.63 | 33.33% | 37.50% | 8.33% | 20.83% |
| UK | 477 | 27.73 | 10.41 | 40.46% | 7.55% | 38.16% | 13.84% |
| Ukraine | 1,122 | 23.53 | 8.42 | 43.32% | 19.96% | 21.03% | 15.69% |
| USA | 2,165 | 36.27 | 14.42 | 33.12% | 7.58% | 21.34% | 37.97% |

Table S2. Reliability coefficient (McDonald's ω) of the ASR-YWMS used in the study across countries.

|  | ω |
| --- | --- |
| All | .93 |
| Argentina | .95 |
| Canada | .93 |
| Chile | .92 |
| Estonia | .92 |
| France | .92 |
| Germany | .93 |
| Hungary | .93 |
| Iran | .86 |
| Italy | .93 |
| Japan | .92 |
| Mexico | .92 |
| Morocco | .72 |
| Netherlands | .71 |
| Nigeria | .95 |
| Philippines | .92 |
| Russia | .93 |
| Serbia | .93 |
| Turkey | .94 |
| Uganda | .94 |
| UK | .96 |
| Ukraine | .92 |
| USA | .92 |

*Note*: ASR-YWMS = Acceptance of Sugar Relationships in Young Women and Men Scale.

Table S3.A. Descriptive statistics of individual-level variables across countries

| Country | ASR-YWMS | | Social media use | |
| --- | --- | --- | --- | --- |
|  | Mean | *SD* | Mean | *SD* |
| All | .15 | 1.02 | 153.89 | 96.46 |
| Argentina | .00 | 1.02 | 149.56 | 88.74 |
| Canada | .49 | 1.12 | 134.22 | 94.16 |
| Chile | .27 | 1.09 | 181.42 | 88.65 |
| Estonia | .19 | .97 | 128.20 | 82.32 |
| France | .00 | .94 | 141.04 | 82.59 |
| Germany | -.04 | .98 | 120.22 | 77.52 |
| Hungary | -.09 | .94 | 140.46 | 83.83 |
| Iran | .11 | 1.02 | 185.54 | 106.38 |
| Italy | -.02 | .95 | 135.61 | 81.15 |
| Japan | .15 | .89 | 119.47 | 100.97 |
| Mexico | .49 | 1.04 | 175.91 | 90.39 |
| Morocco | .72 | .39 | 92.29 | 43.42 |
| Netherlands | .02 | 1.01 | 104.60 | 82.62 |
| Nigeria | .20 | 1.09 | 136.86 | 94.16 |
| Philippines | .64 | 1.11 | 230.68 | 110.11 |
| Russia | .29 | 1.03 | 189.42 | 106.36 |
| Serbia | -.14 | .95 | 144.18 | 84.13 |
| Turkey | -.17 | .93 | 161.50 | 92.33 |
| Uganda | .13 | 1.08 | 149.26 | 107.11 |
| UK | .34 | 1.05 | 141.99 | 89.92 |
| Ukraine | .29 | 1.00 | 177.46 | 99.05 |
| USA | .51 | 1.15 | 132.80 | 95.80 |

Table S3.B. Descriptive statistics of country-level variables across countries

| Country | Cultural tightness-looseness | Internet penetration | Smartphone penetration | Gender wage gap | Female youth unemployment | Net freedom |
| --- | --- | --- | --- | --- | --- | --- |
| All | 56.61(26.95) | 79.57 (20.1) | 123.04 (25.59) | 50.11 (14.66) | 17.04 (9.34) | 64.82 (20.93) |
| Argentina | 75.00 | 87.15 | 130.35 | 50.26 | 26.69 | 71.00 |
| Canada | 84.60 | 93.94 | 87.41 | 60.63 | 12.54 | 86.00 |
| Chile | 86.80 | 90.23 | 136.57 | 46.58 | 22.59 | 86.00 |
| Estonia | 55.40 | 90.98 | 148.74 | 57.78 | 14.93 | 92.00 |
| France | 99.60 | 86.10 | 113.95 | 51.59 | 19.31 | 76.00 |
| Germany | 82.90 | 91.43 | 127.12 | 55.05 | 6.30 | 77.00 |
| Hungary | 42.80 | 88.64 | 105.14 | 52.30 | 15.45 | 69.00 |
| Iran | 31.50 | 78.60 | 153.62 | 13.09 | 36.57 | 12.00 |
| Italy | 67.80 | 74.86 | 130.78 | 39.88 | 32.85 | 75.00 |
| Japan | 42.30 | 82.91 | 159.52 | 53.52 | 4.15 | 78.00 |
| Mexico | 74.40 | 75.63 | 99.09 | 44.62 | 8.32 | 61.00 |
| Morocco | .00 | 88.13 | 138.91 | 20.70 | 25.87 | 54.00 |
| Netherlands | 78.90 | 92.05 | 123.45 | 61.80 | 8.96 | 83.00 |
| Nigeria | 17.90 | 35.51 | 89.29 | 77.08 | 10.50 | 59.00 |
| Philippines | 31.50 | 66.91 | 144.42 | 44.20 | 10.19 | 60.00 |
| Russia | 57.20 | 88.21 | 169.07 | 55.17 | 17.39 | 20.00 |
| Serbia | 61.80 | 81.17 | 125.08 | 49.45 | 27.14 | 70.00 |
| Turkey | 12.50 | 81.41 | 99.54 | 32.77 | 28.27 | 31.00 |
| Uganda | 37.40 | 10.00 | 65.62 | 74.54 | 6.35 | 53.00 |
| UK | 89.30 | 96.20 | 117.89 | 58.00 | 11.17 | 78.00 |
| Ukraine | 56.90 | 79.22 | 135.03 | 47.74 | 20.43 | 59.00 |
| USA | 58.00 | 91.27 | 106.33 | 55.63 | 8.85 | 76.00 |

*Note*. Standard deviations (numbers in the parentheses) were calculated only for the whole dataset.
